# Early Outcomes and Risk Factors in Orthognathic Surgery for Mandibular and Maxillary Hypo- and Hyperplasia: A 13-Year Analysis of a Multi-Institutional Database

**DOI:** 10.3390/jcm12041444

**Published:** 2023-02-11

**Authors:** Samuel Knoedler, Helena Baecher, Cosima C. Hoch, Doha Obed, Dany Y. Matar, Carsten Rendenbach, Bong-Sung Kim, Leila Harhaus, Martin Kauke-Navarro, Gabriel Hundeshagen, Leonard Knoedler, Dennis P. Orgill, Adriana C. Panayi

**Affiliations:** 1 Division of Plastic Surgery, Department of Surgery, Brigham and Women’s Hospital, Harvard Medical School, Boston, MA 02115, USA; 2 Department of Plastic, Hand and Reconstructive Surgery, University Hospital Regensburg, 93053 Regensburg, Germany; 3 Medical Faculty, Friedrich Schiller University Jena, 07737 Jena, Germany; 4 Department of Plastic, Aesthetic, Hand and Reconstructive Surgery, Hannover Medical School, 30625 Hannover, Germany; 5 Department of Oral and Maxillofacial Surgery, Charité—Universitätsmedizin Berlin, Corporate Member of Freie Universität Berlin, Humboldt-Universität zu Berlin, and Berlin Institute of Health, 10117 Berlin, Germany; 6 Department of Plastic Surgery and Hand Surgery, University Hospital Zurich, 8091 Zurich, Switzerland; 7 Department of Hand, Plastic and Reconstructive Surgery, Microsurgery, Burn Trauma Center, BG Trauma Center Ludwigshafen, University of Heidelberg, 67071 Ludwigshafen am Rhein, Germany; 8 Division of Plastic Surgery, Department of Surgery, Yale New Haven Hospital, Yale School of Medicine, New Haven, CT 06520, USA; 9 Division of Plastic and Reconstructive Surgery, Massachusetts General Hospital, Harvard Medical School, Boston, MA 02115, USA

**Keywords:** jaw surgery, orthognathic surgery, mandibular hypoplasia, mandibular hyperplasia, maxillary hypoplasia, maxillary hyperplasia, risk factors

## Abstract

Background: Orthognathic surgery (OS) is a frequently performed procedure for the correction of dentofacial deformities and malocclusion. Research on OS is mostly limited to single-surgeon experience or single-institutional reports. We, therefore, retrospectively analyzed a multi-institutional database to investigate outcomes of OS and identify risk factors for peri- and postoperative complications. Methods: We reviewed the American College of Surgeons National Surgical Quality Improvement Program (ACS-NSQIP) database (2008–2020) to identify patients who underwent OS for mandibular and maxillary hypo- and hyperplasia. The postoperative outcomes of interest included 30-day surgical and medical complications, reoperation, readmission, and mortality. We also evaluated risk factors for complications. Results: The study population included 674 patients, 48% of whom underwent single jaw surgery, 40% double jaw surgery, and 5.5% triple jaw surgery. The average age was 29 ± 11 years, with an equal gender distribution (females: n = 336; 50%, males: n = 338; 50%). Adverse events were relatively rare, with a total of 29 (4.3%) complications reported. The most common surgical complication was superficial incisional infection (n = 14; 2.1%). While the multivariable analysis revealed isolated single lower jaw surgery (*p* = 0.03) to be independently associated with surgical complication occurrence, it also identified an association between the outpatient setting and the frequency of surgical complications (*p* = 0.03) and readmissions (*p* = 0.02). In addition, Asian ethnicity was identified as a risk factor for bleeding (*p* = 0.003) and readmission (*p* = 0.0009). Conclusion: Based on the information recorded by the ACS-NSQIP database, our analysis underscored the positive (short-term) safety profile of OS. We found OS of the mandible to be associated with higher complication rates. The calculated risk role of OS in the outpatient setting warrants further investigation. A significant correlation between Asian OS patients and postoperative adverse events was found. Implementation of these novel risk factors into the surgical workflow may help facial surgeons refine their patient selection and improve patient outcomes. Future studies are needed to investigate the causal relationships of the observed statistical correlations.

## 1. Introduction

Orthognathic surgery (OS) procedures are frequently performed to correct dentofacial deformities and malocclusion. Its principles include surgical manipulation of the bones of the facial skeleton, mainly the maxilla and mandible, in order to restore anatomical relationships and function [1]. OS is indicated for patients with moderate or severe medical conditions that exceed the capabilities of orthodontics [2,3]. While functional problems and malocclusion are considered the main reasons for seeking OS, this type of procedure may also be used to manage pain-related maxillofacial problems, such as temporomandibular joint disorders [3,4]. By aligning the patient’s jaw into a healthy position, OS can help correct skeletal disharmonies and asymmetries. The resulting improvements in dentofacial aesthetics play a major role in patient satisfaction and during surgical decision-making [5,6,7].

When preparing and determining the surgical plan, OS-associated risks must be carefully taken into account. Therefore, the identification of complication predictors is essential for perioperative risk profile assessment. Potential adverse events of OS are far-reaching, ranging from hemorrhage and excessive bleeding through infection to unwanted fracture, bad split, or bone necrosis. Complication rates vary widely across the literature, with overall complication rates ranging from 4 to 27% [1,4,8,9,10,11,12,13,14,15,16,17]. This variation is partly due to inconsistent reporting of complications, ranging from minor dental defects to more serious complications, such as bleeding. According to various studies described over the past few years, sex, age, presence or absence of third molars, surgery duration, surgeon experience, type of maxillomandibular deformity, and single jaw or bimaxillary orthognathic surgery are risk factors for complications [16,18,19,20,21].

Most of the evidence on the complication rates of orthognathic surgery procedures is derived from single-institution series with small sample sizes, which might lead to bias and limit the research significance and transferability. With this parameter distribution model, complication rates of patients undergoing OS are often not reported in academic publications. However, consistent statistics of complications and their predictors are essential for perioperative risk assessment and patient counseling. Analyses of multi-center databases help overcome such limitations and limit bias by integrating patient data with geographic and institutional differences. Using such a multi-institutional database in the context of OS would allow us to identify more robust risk factors and provide a comprehensive overview of postoperative outcomes in this diverse patient cohort.

The National Surgical Quality Improvement Program of the American College of Surgeons (NSQIP) collects validated data from more than 700 US hospitals, resulting in a large and diverse patient collection. Specifically, in the field of oral and maxillofacial surgery, the current literature includes numerous studies evaluating the NSQIP data [22,23,24,25,26,27,28,29,30,31]. Accordingly, analyses of the NSQIP records can provide valuable and insightful information that facial surgeons may wish to implement into their perioperative workflow. To the best of our knowledge, the NSQIP data have not been analyzed to determine OS outcomes in this study profile. This analysis aimed to fill this research gap by querying the ACS-NSQIP database and identifying the most common types of complications associated with OS and their associations with possible risk factors. These data can ultimately help facial surgeons performing OS make informed decisions considering the morbidity of the procedures.

## 2. Materials and Methods

### 2.1. Data Source and Patient Selection

Data were gathered over a 13-year period between 2008 and 2020 from the American College of Surgeons National Surgical Quality Improvement Program (ACS-NSQIP) database. Developed by the American College of Surgeons, the ACS-NSQIP database represents a multi-institutional and risk-adjusted data collection of surgical patients and procedures, available to participating institutions. Quality, reliability, and validity of the database are warranted by spot audits and peer controls. Institutional Review Board (IRB) approval (Protocol #: 2013P001244) was obtained from our institution (Brigham and Women’s Hospital, Boston, MA, USA).

The ACS-NSQIP catalog was queried between 2008 and 2020 to identify all patients who underwent OS procedures. A total of 13 annual data sets were filtered by the codes ICD-9-CM 524.01 (“Major anomalies of jaw size, maxillary hyperplasia”), 524.02 (“Major anomalies of jaw size, mandibular hyperplasia”), 524.03 (“Major anomalies of jaw size, maxillary hypoplasia”), 524.04 (“Major anomalies of jaw size, mandibular hypoplasia”), and ICD-10-CM M26.01 (“Maxillary hyperplasia”), M26.02 (“Maxillary hypoplasia”), M26.03 (“Mandibular hyperplasia”), and M26.04 (“Mandibular hypoplasia”). Patients with other and/or more far-reaching diagnoses, such as syndromes with dentofacial manifestations, were not included. All cases with physiologically impossible body mass indices (<7 kg/m^2^ or >250 kg/m^2^) were excluded as miscoding. Patients under 18 years of age were not eligible. Any cases with treatments reaching beyond the OS scope and/or concurrent non-OS interventions were excluded. The generated patient pool was manually reviewed by two investigators (SK and AP), and the classification as OS was verified for each case. A third investigator (LK) was consulted in order to resolve any discrepant assessments. As a result, we compiled a homogenous cohort of patients who had been diagnosed with mandibular or maxillary hypo- or hyperplasia and underwent OS.

### 2.2. Variable Extraction

Pre-, peri-, and 30-day postoperative variables were extracted.

(i) Preoperative data included patient demographics (gender, age, race), comorbidities (diabetes mellitus [insulin-dependent or not], chronic obstructive pulmonary disease [COPD], obesity [body mass index above 30], active dialysis treatment, hypertension, dyspnea, smoking status, corticosteroid use, disseminated cancer, and wound infection), as well as preoperative scores (the American Society of Anesthesiology (ASA) physical status classification [score 1–4] and wound classification [score 1–4]). Moreover, we evaluated the functional health status (independent versus partially or totally dependent) and calculated the body mass index using the formula “[weight (pounds)/height (inches)^2^ × 703]”. All extracted preoperative parameters are listed in Table 1. Further, the specific preoperative diagnoses were excerpted and classified according to the underlying ICD-9-CM and ICD-10-CM codes. Table 2 provides a detailed breakdown of the preoperative diagnoses.
(ii) In terms of perioperative variables, we analyzed the surgical specialty (otolaryngology, plastic surgery, general surgery, and others), the type of anesthesia (general, monitored anesthesia care, epidural/spinal), and the year of surgery. Additionally, we specified the setting differentiating between in- and outpatient care. The surgical characteristics are displayed in Table 3. For in-depth assessment, we manually classified all cases into single, double, and triple jaw (i.e., the combination of double jaw with concurrent genioplasty) surgeries. To further refine this classification pattern, we specified the operated jaw and identified all patients that underwent concurrent intranasal procedures. This (sub)classification scheme was manually reviewed and independently verified by two investigators (LK and HB). In some cases (“Others”), a more detailed definition of the performed procedure (e.g., “Osteoplasty” or “Osteotomy”) was not applicable due to the limited case information captured. When classifying and specifying the (sub)types of surgery, we closely followed the nomenclature entered in the ACS-NSQIP database. The classification and frequency of the specific types of surgery are summarized in Table 3.
(iii) As 30-day postoperative outcomes, we evaluated the operating time, the length of hospital stay (LOS), and the destination of discharge (home, other/unknown). LOS was calculated as the difference in days between the date of admission and the date of discharge. Any complication was defined as the occurrence of either patient mortality and/or reoperation and/or readmission and/or unplanned readmission and/or any surgical and/or any medical complication. All surgical complications that are captured in the ACS-NSQIP database and occurred at least once were analyzed (i.e., superficial and deep incision site infections, organ space infections, and bleeding). Likewise, while considering all medical complications documented in the ACS-NSQIP database, we concentrated on those in which at least one case has been reported (i.e., reintubation, infection of the urinary tract, and deep vein thrombosis/thrombophlebitis). The (post)operative outcomes following OS are shown in Table 4 and Table 5. Table 6 provides a detailed breakdown of all cases with any complications.

### 2.3. Statistical Analysis

Data were collected and saved in an electronic laboratory notebook (LabArchives, LLC, San Marcos, CA, USA), and evaluated using GraphPad Prism (V9.00 for macOS, GraphPad Software, La Jolla, CA, USA). Analyzed with independent *t*-tests, continuous variables are recorded as means with standard deviations. To measure differences in categorical variables, Pearson’s Chi-square was applied. In cases with fewer events than 10, Fisher’s exact test was applied. The threshold for statistical significance was set at *p* < 0.05. To identify risk factors for complications, univariable subgroup analysis was carried out, partitioning the cohort into three groups depending on the occurrence of any surgical and medical complications. To eliminate confounding factors, multivariable regression was performed by including all variables found to be significant predictors of the occurrence of any complication.

## 3. Results

### 3.1. Patient Demographics and Diagnoses

The study population included 674 patients who underwent OS over a 13-year review period (2008–2020). The mean patient age and BMI were 29 ± 11 years and 26 ± 5.6 kg/m^2^, respectively. Caucasian patients (n = 450; 67%) represented the majority of our patient cohort, and proportions were equally distributed among the genders (females: n = 336; 50%, males: n = 338; 50%). While obesity (i.e., a BMI over 30; n = 128; 19%) was the most prevalent comorbidity, 7% (n = 47) of patients were current smokers. Detailed demographic data and comorbidities are shown in Table 1. Maxillary hypoplasia was proportionally the most common preoperative diagnosis, accounting for 57% (n = 387) of cases. While about one-fourth (n = 161; 24%) of the patients were diagnosed with mandibular hypoplasia, 89 patients (13%) suffered preoperatively from mandibular hyperplasia. In 5.5% (n = 37) of cases, maxillary hyperplasia was treated surgically with OS. Table 2 provides an overview of the preoperative diagnoses.

### 3.2. Surgical Characteristics

**Figure 1:**
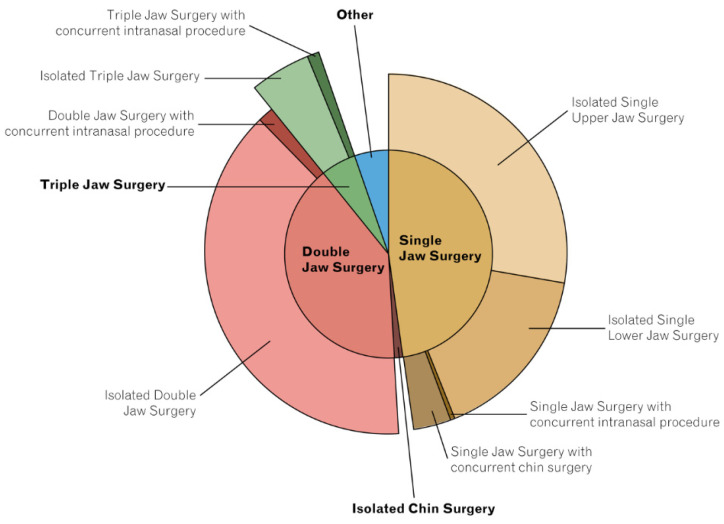
Procedure distribution. The majority of patients underwent single jaw surgery (47.8%), followed by double jaw surgery (40.1%). Other procedures, which constituted 5.3% of all procedures included, for example, unspecified osteoplasties and combined genioplasty plus intranasal procedures.

Almost 100% (n = 672) of OS took place under general anesthesia, with otorhinolaryngologists performing the majority of OS (n = 556; 82%) procedures. While the majority of OS procedures were performed in an outpatient setting (n = 411; 61%), 263 cases (39%) were treated as inpatients (Table 3). Single jaw surgery accounted for 48% (n = 322), with isolated single upper jaw surgery performed in 28% (n = 187) and isolated single lower jaw surgery in 16% (n = 109) of cases. The proportion of double jaw surgery was 40% (n = 270), while 37 patients (5.5%) received triple jaw surgery (Figure 1; Table 3).

### 3.3. Perioperative Outcomes and Postoperative Surgical and Medical Outcomes

Mean operation time was 183± 115 min, and postoperative LOS was 0.9 ± 4.0 days on average, with 93% (n = 627) of patients discharged home afterward (Table 4). Within the postoperative period of 30 days, no case of death occurred, and five (0.7%) patients returned to the operating room. Any complications, i.e., reoperation, readmission, and surgical or medical complication, were reported in 4.3% (n = 29) of patients. Further details are shown in Table 5 and Table 6. The surgical complication rate was 2.8% (n = 19), with superficial incisional infection (n = 14; 2.1%) as the most common surgical complication. Medical adverse events accounted for 0.7% (n = 5) of cases.

In univariable analysis, outpatient setting (*p* = 0.008) was identified as a risk factor for the occurrence of surgical complications (Table 7). Multivariable analysis confirmed the outpatient setting as an independent risk factor for the occurrence of any surgical complication (*p* = 0.03) and readmission (*p* = 0.02). Patients with insulin-treated diabetes were at significantly higher risk for returning to the operating room (*p* < 0.0001). Similarly, Asian race was found to be a significant risk factor, not only for reoperation (*p* = 0.0009) but also for required postoperative blood transfusions (*p* = 0.003). A significant positive correlation between isolated single lower jaw surgery and the occurrence of any surgical complications (*p* = 0.03) and, more specifically, superficial incisional infection (*p* = 0.04) was noted. Further details on the multivariable risk factor assessment are displayed in Table 8.

## 4. Discussion

Heterogenicity in the type of OS procedure (i.e., mandibular osteotomy, LeFort I osteotomy, or bimaxillary osteotomy, with modifications in segmentation), technical modifications, and level of skeletal discrepancies necessitate comprehensive data pools for valid investigation of adverse side effects [32,33]. Multi-institutional databases, such as the ACS-NSQIP, are predestined to overcome these discrepancies and represent powerful tools for evaluating the generalizable pattern of perioperative workflow.

### 4.1. Safety of OS

Strikingly, not a single OS-associated death was reported during the 30-day postoperative follow-up. This non-existent mortality risk in combination with the overall low complication rate of 4.3% suggests OS to be relatively safe. Our findings are consistent with the current body of evidence. While Olate et al. and Glen et al. also documented no deaths associated with OS, Ferri et al. and Bacos et al. reported complication rates of 1.5% and 4.5%, respectively [20,32,33,34]. Notably, during the 30-day postoperative follow-up, no case of pulmonary embolism, unplanned reintubation, prolonged ventilator dependence, renal disorder, cerebral vascular accident, cardiac infarction/arrest, or sepsis/septic shock has been reported. Furthermore, no patient suffered postoperatively from wound dehiscence (Table 4). In this context, it is worth mentioning that the patient cohort undergoing OS typically consists predominantly of young and physically healthy candidates. In our study population, the average patient was under 30 years, had a BMI of 26 (numbers below the U.S. national average), and very rarely suffered from comorbidities [35].

### 4.2. Mandible as Risk Center for Surgical Complications

It is well known that different surgical procedures in the field of OS are associated with varying complication rates [21]. In agreement with the available literature, our findings comprise a significantly higher occurrence of any surgical complication in isolated single lower jaw surgery when compared to other types of corrective jaw surgery (Table 8) [1,10,17]. Special anatomical conditions of the mandible bone, such as its masticatory muscle attachment and articulatory function, perioperative difficulties in the visualization of the inferior alveolar neurovascular bundle, and sophisticated osteotomy and fixation techniques, are factors that may contribute to a more frequent occurrence of adverse events [36]. Moreover, the lower jaw is more susceptible to pseudoarthrosis, which is outlined by an analysis of Ferri et al., where mandibular pseudoarthrosis arose in 4 of 5025 cases (<0.1%) [32]. In terms of surgical site infections (SSI) associated with OS, Cousin et al. reported in a single-institutional study of 512 cases an infection rate of 8%, with 93% located on the mandibular and 7.3% on the maxillary site [37]. Furthermore, reviewing 2910 single-institutional OS cases, Chow et al. found SSIs in 7.4% of patients, equally distributed between the mandible and maxilla [8]. Low infection rates of 1.2%, exclusively affecting the mandible, were reported by Ferri et al. [32]. By comparison, we found SSI rates of 2.7%, indicating a moderate complication rate when averaged over multiple institutions (Table 4). Strikingly, in multivariable analysis, isolated single lower jaw surgery was found to be significantly predestined for higher SSI occurrence. Such higher infection rates in mandibular procedures may be caused by lower blood supply in the mandible (as compared to the maxilla) and bacteria-rich salivary stasis in the lower jaw region due to gravitation forces [38].

### 4.3. Outpatient OS Surgery as a Risk Factor for Complications

Due to the need for cost control in combination with high-quality treatment options, outpatient OS is of increasing popularity [39]. This trend could be confirmed in our patient sample, with approximately two-thirds of OS being performed in an outpatient setting. However, in our study, outpatient procedures accounted for almost 90% of surgical adverse events (Table 7). Accordingly, the multivariable analysis suggested a significant correlation between the outpatient setting and the occurrence of any surgical complication and readmission (Table 8).

This finding is consistent with previous reports: while Kantar et al. found that OS patients undergoing bimaxillary interventions in an outpatient setting had an almost tenfold higher risk of developing wound complications, Knoff et al. reported elevated admission rates of more than 16% throughout their 9-year experience with outpatient OS [21,40]. However, this statistically significant correlation seems to be mainly due to one specific year, as half of all 20 complication cases in the outpatient setting (10/20) occurred in 2014. Due to escalating health care costs and limited reimbursement from insurance plans, OS patients increasingly considered the more cost-effective outpatient surgery during the early and mid-2010 decade [41,42,43]. This shift to outpatient care is also reflected in our analysis, with a skyrocketing number of outpatient OS in 2014: in this year, we recorded a total of 118 OS procedures, of which 85% (n = 100) were performed in the outpatient setting. Compared to 2013 (n = 18 outpatient procedures), this implies a more than 8-fold increase in outpatient OS load. Particularly in the field of oral and maxillofacial surgery (OMFS), outpatient surgical care must be “at a level equal to or [even] superior to that offered within the hospital environment” [41]. While Berenstein et al. and Hattori et al. concluded that outpatient OS may be performed safely in principle, they also underscored the need for multimodal perioperative management, with adequate patient preparation and close one-to-one nursing in the recovery room [44,45]. Similarly, Dann emphasized that thorough postoperative surveillance was an essential component of his OMFS practice transition from the inpatient model to a surgicenter focusing on outpatient procedures [46]. One might hypothesize that the 2014 surge of outpatient OS has (over)strained the standard of care, with less close-knit patient control and subsequently higher complication rates. Of note, given the trend of decreasing reimbursement by insurance companies at that time, many surgeons have changed their formerly orthognathic practices to parallel that of a cosmetic office (exempt from insurance constraints). It is, therefore, not surprising that between 2014 and 2016, criticisms arose about inadequate preoperative patient screening and lack of intervention options for perioperative adverse events in the outpatient setting, potentially jeopardizing patient safety [41,47,48,49]. These concerns were also evident in a 2014 report revealing that most surgeons—despite the escalated treatment cost—still considered the hospital setting to be the most appropriate for OS [41]. Emerging OS-related operative trends may have also contributed to the peak complication rates in 2014. Indeed, during this period, the “surgery-first approach” has gained popularity, and novel digital tools, such as advanced three-dimensional medical imaging, computer-aided design, and computer-aided manufacturing, have paved their way into the surgical OS workflow [50,51]. Naturally, these novelties require gradual familiarization and might be associated with increased complication rates in their early application stages.

While the underlying rationale for the 2014 complication spike cannot be fully understood, our findings reinforce the call for fine-tuned preoperative planning and critical evaluation of the patient’s eligibility for ambulatory OS. Of note, there is ongoing research aiming to further investigate the risk profile of outpatient OS and decipher the factors associated with patient hospitalization [52].

### 4.4. Racial Risk Disparities among OS Patients

Investigating racial disparities in the occurrence of OS-related postoperative complications, Asian patients showed a significantly higher risk of surgical complications (Table 6 and Table 7). Multivariable analysis confirmed such racial risk correlation, with statistically significant associations between Asian OS patients and the need for readmission and blood transfusion (Table 8). Notably, when analyzing the occurrence of surgical complications among different races, Pollack et al. also found Asian patients to be at higher risk of hemorrhage [53]. Misumida et al. reiterated the disproportionate risk of bleeding in Asian patients, reporting a significant correlation between Asian ethnicity and the occurrence of major in-hospital bleeding [54]. During treatment with antiplatelets, anticoagulants, and thrombolytic agents, this proneness to bleeding was found to exacerbate, with an incidence of intracranial hemorrhage four times higher and general bleeding events occurring twice as frequently in Asians [55,56,57,58]. Reviewing cases of surgical palate cleft repair, Wu et al. reported increased rates of accidental puncture and fistula among Asian patients [59]. In Asian patients, variances in metabolism and anatomy, such as bimaxillary protrusion, shorter mandibles, as well as a deviated course of the maxillary artery, may lead to different predispositions for surgical risks [60,61,62,63]. Interestingly, among a subset of OS patients, Asian ethnicity was associated with significantly prolonged LOS and markedly higher therapy costs (when compared to Caucasians)—suggesting a higher complexity of OS in Asian patients [64]. To the best of our knowledge, this is the first study to outline the ethnic divergence in surgical outcomes associated with OS and reveal an increased risk of bleeding among OS Asian patients. However, it is important to note that our statistical findings in this context are limited to a small number of patients and should, therefore, be interpreted with caution. More precisely, in our analysis, a total of 71 Asian patients were included, 6 (6/71; 8.5%) of whom experienced a surgical complication. These six cases account for nearly one-third of all reported surgical adverse events (6/19; 31.6%). Four bleeding incidents were recorded, all of which occurred in Asian patients (4/4; 100%). Given the overall small number of patients, these statistical observations should be understood primarily as signals that warrant an in-depth and comprehensive investigation. Future large-scale studies are needed to further validate the risk predisposition of Asian OS patients.

The majority of existing studies evaluating complication risks of OS are limited to single-institution analysis. As in current works, data were collected from one single surgical center. They are not complying with the heterogeneity of surgical and medical conditions between different institutions [33,36,65], not to mention reports of single-surgeon experiences, which describe in general lower complication rates, compared to single- and multi-institutional studies [17,32,66]. While the few multi-institutional studies focus on several subgroups, such as type of surgery or elderly patients, we try to fill the gap of comprehensive investigations in OS, adjusted to state-to-the-art surgical techniques [20,21,67].

### 4.5. Limitations

This study is the first to analyze risk factors and early outcomes in orthognathic surgery for mandibular and maxillary hypo- and hyperplasia—based on multi-institutional and diversified data collected over 13 years. Nevertheless, when interpreting the findings and drawing research conclusions, its limitations should be carefully considered. General limitations include the retrospective nature of the ACS-NSQIP database associated with inherent biases and confounders. Further, the accuracy and quality of the information entered depend on subjective assessment and practical knowledge [68]. The multi-institutional (and multi-surgeon) extent of the database has been suggested as a potential root of bias, as the quality may vary both within and between the participating institutions. Variance in the overall standard of care between the NSQIP hospitals and differences in surgeons’ skills and expertise are factors that are known to affect perioperative outcomes. However, when evaluating the data quality and interrater-reliability of the ACS-NSQIP database, Shiloach et al. identified low variance in the database’s heterogeneity [69]. It should also be noted that the specialty of oral and maxillofacial surgery is not listed separately in the NSQIP database. Yet, the existence and value of various NSQIP-based studies published in the field of oral and maxillofacial surgery relativize this potential limitation. The standardized data capture leads to a lack of procedure-specific information. For example, the type of osteosynthesis and the mode of surgical planning (conventional versus virtual) are not specified. In addition, the catalog misses intraoperative information on short-term (<30 days) procedure-specific complications, such as hematoma and dental/periodontal complications. Certain long-term (>30 days) procedure-specific outcomes, for example, nerve injury, bone non-union, aesthetics, and functionalities are not available. The overall number of complications is relatively low, which limits the significance and generalizability of our results; therefore, conclusions should be drawn with caution. In addition, it should be emphasized that we report only statistical correlations and not causal relationships. The underlying causalities of the observed correlations need to be investigated in future studies.

## 5. Conclusions

By analyzing 674 cases undergoing OS, we corroborated the general low complication risk in OS performed across multiple institutions. We found that lower jaw surgery was associated with an increased risk of perioperative complications. Our statistical calculations also revealed a positive correlation between outpatient OS care and the occurrence of adverse events which may be primarily due to a peak in complications rates in 2014. In addition, Asian OS patients are at a potentially increased risk for postoperative adverse events. These evidence-based findings may help facial surgeons to refine their OS patient selection and identify risk candidates in the preoperative planning stage. Our findings warrant further investigations, with future studies being needed to decipher the underlying causalities of the statistical correlations presented.

## Figures and Tables

**Table 1:** Patient demographics and comorbidities. Reported as n (%).

Characteristic	Jaw Surgery (n = 674)
**Demographics**	
Sex	
Female (n)	336 (50)
Male (n)	338 (50)
Age, mean ± SD	29 ± 11
BMI, mean ± SD	26 ± 5.6
**Race**	
American Indian or Alaskan native	2 (0.3)
Asian	71 (11)
Native Hawaiian or Pacific Islander	0 (0.0)
Black or African American	53 (7.9)
White	450 (67)
Other or unknown	98 (15)
**Preoperative health and comorbidities**	
Diabetes	10 (1.5)
Insulin treated diabetes	4 (0.6)
COPD	1 (0.1)
Obesity	128 (19)
Dialysis	1 (0.1)
Hypertension	26 (3.9)
Dyspnea	3 (0.4)
Current smoker	47 (7.0)
Corticosteroid use	7 (1.0)
Disseminated cancer	1 (0.1)
Wound infection	1 (0.1)
**ASA class**	
1—No disturbance	359 (53)
2—Mild disturbance	294 (44)
3—Severe disturbance	21 (3.1)
4—Life-threatening	0 (0.0)
**Wound class**	
1—Clean	44 (6.5)
2—Clean/Contaminated	623 (92)
3—Contaminated	7 (1.0)
4—Dirty/Infected	0 (0.0)
**Functional Status**	
Independent	673 (100)
Partially or Totally Dependent	0 (0.0)

**Table 2:** Preoperative diagnoses according to ICD-9-CM and ICD-10-CM. Reported as n (%).

Diagnoses	Jaw Surgery (n = 674)
Mandibular Hypoplasia	161 (24)
Mandibular Hyperplasia	89 (13)
Maxillary Hypoplasia	387 (57)
Maxillary Hyperplasia	37 (5.5)

**Table 3:** Surgical characteristics. Reported as n (%).

Characteristic	Jaw Surgery (n = 674)
**Type of Surgery**	
**Single Jaw Surgery**	322 (47.8)
Isolated Single Upper Jaw Surgery	187 (27.7)
Isolated Single Lower Jaw Surgery	109 (16.2)
Single Jaw Surgery with concurrent intranasal procedure	3 (0.4)
Single Jaw Surgery with concurrent genioplasty	23 (3.4)
**Isolated Genioplasty**	9 (1.3)
**Double Jaw Surgery**	270 (40.1)
Isolated Double Jaw Surgery	260 (38.6)
Double Jaw Surgery with concurrent intranasal procedure	10 (1.5)
**Triple Jaw Surgery**	37 (5.5)
Isolated Triple Jaw Surgery	31 (4.6)
Triple Jaw Surgery with concurrent intranasal procedure	6 (0.9)
**Other**	36 (5.3)
**Surgical Specialty**	
Otolaryngology	556 (82)
Plastics	84 (12)
General Surgery	29 (4.3)
Other	5 (0.7)
**Type of anesthesia**	
General	672 (100)
Other/Unknown	2 (0.3)
**Year of surgery**	
2008	11 (1.6)
2009	14 (2.1)
2010	22 (3.3)
2011	5 (0.7)
2012	28 (4.2)
2013	28 (4.2)
2014	118 (18)
2015	117 (17)
2016	111 (16)
2017	77 (11)
2018	31 (4.6)
2019	69 (10)
2020	43 (6.3)
**Setting**	
Inpatient	263 (39)
Outpatient	411 (61)

**Table 4:** Operative and postoperative outcomes following jaw surgery. Reported as n (%), unless otherwise stated.

Outcome	Jaw Surgery (n = 674)
Length of Hospital Stay, Mean days ± SD	0.9 ± 4.0
Operative time, Mean minutes ± SD	183 ± 115
**Any Complication**	29 (4.3)
Mortality within 30 days	0 (0.0)
Reoperation	5 (0.7)
Readmission	5 (0.7)
Unplanned Readmission	5 (0.7)
**Surgical Complication**	19 (2.8)
Superficial Incisional Infection	14 (2.1)
Deep Incisional Infection	3 (0.4)
Organ Space Infection	2 (0.3)
Bleeding	4 (0.6)
Dehiscence	0 (0.0)
**Medical Complication**	5 (0.7)
Reintubation	2 (0.3)
Urinary Tract Infection	2 (0.3)
Deep Vein Thrombosis/Thrombophlebitis	1 (0.1)
Pulmonary Embolism	0 (0.0)
Unplanned Reintubation	0 (0.0)
Pneumonia	0 (0.0)
Ventilator Dependence > 48 h	0 (0.0)
Progressive Renal Insufficiency	0 (0.0)
Acute Renal Failure	0 (0.0)
Stroke/Cerebral Vascular Accident	0 (0.0)
Cardiac Arrest	0 (0.0)
Myocardial Infarction	0 (0.0)
Sepsis	0 (0.0)
Septic Shock	0 (0.0)
**Discharge destination**	
Home	627 (93)
Other/unknown	47 (7.0)

**Table 5:** Distribution of procedures with the type-specific occurrence of any complication.

Type of Surgery	Total	Any Comp	Any Comp/Total%
**Single Jaw Surgery**	322	14	4.3
Isolated Single Upper Jaw Surgery	187	7	3.7
Isolated Single Lower Jaw Surgery	109	6	5.5
Single Jaw Surgery with concurrent intranasal procedure	3	0	0.0
Single Jaw Surgery with concurrent genioplasty	23	1	4.3
**Isolated Genioplasty**	9	0	0.0
**Double Jaw Surgery**	270	12	4.4
Isolated Double Jaw Surgery	260	11	4.2
Double Jaw Surgery with concurrent intranasal procedure	10	1	10
**Triple Jaw Surgery**	37	1	2.7
Isolated Triple Jaw Surgery	31	1	3.2
Triple Jaw Surgery with concurrent intranasal procedure	6	0	0.0
**Other**	36	2	5.5

**Table 6:** Detailed information on all cases with complications. Adverse events occurred in 29 cases (4.3%). All readmissions were unplanned.

	Race	Reoperation	Readmission	Surgical Complication	Medical Complication
**Isolated Single Upper Jaw Surgery**					
Male, 23 Years	Caucasian	X			
Female, 29 Years	Caucasian				1 (Reintubation)
Male, 42 Years	Caucasian			1 (SSI)	
Female, 18 Years	Unknown				1 (Reintubation)
Male, 23 Years	Caucasian			1 (SSI)	
Female, 26 Years	Asian	X		1 (Bleeding)	
Female, 56 Years	Caucasian				1 (Urinary Tract Infection)
**Isolated Single Lower Jaw Surgery**					
Female, 20 Years	Caucasian			1 (SSI)	
Female, 55 Years	Caucasian			1 (SSI)	
Male, 20 Years	Asian			2 (SSI + Bleeding)	
Female, 32 Years	Caucasian	X	X		
Male, 36 Years	Caucasian			2 (SSI + OSI)	
Male, 24 Years	Asian			1 (SSI)	
**Single Jaw Surgery with concurrent genioplasty**					
Female, 19 Years	Asian		X	2 (OSI + Bleeding)	
**Isolated Double Jaw Surgery**					
Male, 20 Years	Unknown		X		
Male, 29 Years	Caucasian	X			
Female, 39 Years	Caucasian			1 (SSI)	
Male, 19 Years	Asian			1 (DII)	
Male, 33 Years	Caucasian			1 (SSI)	
Female, 20 Years	Caucasian				1 (Urinary Tract Infection)
Male, 39 Years	Caucasian			1 (SSI)	
Male, 22 Years	Asian		X	2 (SSI + Bleeding)	
Female, 38 Years	Caucasian			1 (SSI)	
Male, 43 Years	Unknown			1 (DII)	
Female, 40 Years	Caucasian			1 (SSI)	
**Double Jaw Surgery with concurrent intranasal procedure**					
Female, 26 Years	Caucasian			1 (DII)	
**Isolated Triple Jaw Surgery**					
Female, 20 Years	Caucasian			1 (SSI)	
**Others**					
*including each procedure entered*					
Mandibular Reconstruction + Mandibulectomy + Free Flap Reconstruction (Male, 61 Years)	Caucasian				1 (Deep Vein Thrombosis)
Mandibular Osteotomy + Free Skin Flap (Male, 42 Years)	Caucasian	X	X		

SSI: superficial incisional infection; OSI: organ space infection; DII: deep incisional infection.

**Table 7:** Risk factors for complications. Reported as n (%), unless otherwise stated. Statistically significant *p* values are highlighted in bold.

	Any Complication			Surgical Complication			Medical Complication		
Characteristic	Yes (n = 29)	No (n = 645)	*p* Value	Yes (n = 19)	No (n = 655)	*p* Value	Yes (n = 5)	No (n = 669)	*p* Value
**Demographics**									
**Sex**			0.58			>0.99			>0.99
Female	13 (45)	323 (50)		9 (47)	327 (50)		2 (40)	334 (50)	
Male	16 (55)	322 (50)		10 (53)	328 (50)		3 (60)	335 (50)	
Age, mean ± SD	32 ± 12	29 ± 11		31 ± 11	29 ± 11		37 ± 3	29 ± 11	
BMI, mean ± SD	25 ± 6	26 ± 6		26 ± 6	26 ± 6		22 ± 3	26 ± 6	
**Race**			0.21			**0.011**			0.88
American Indian/Alaskan native	0 (0.0)	2 (0.3)		0 (0.0)	2 (0.3)		0 (0.0)	2 (0.3)	
Asian	6 (21)	65 (10)		6 (32)	66 (10)		0 (0.0)	71 (11)	
Black/African American	0 (0.0)	53 (8.2)		0 (0.0)	53 (8.1)		0 (0.0)	53 (7.9)	
White	20 (69)	430 (67)		12 (63)	437 (67)		4 (80)	446 (67)	
Other or unknown	3 (10)	95 (15)		1 (5.3)	97 (15)		1 (20)	97 (14)	
**Setting**			0.44			**0.008**			0.38
Outpatient	20 (69)	391 (61)		17 (89)	394 (60)		2 (40)	409 (61)	
Inpatient	9 (31)	254 (39)		2 (11)	261 (40)		3 (60)	260 (39)	
**Preop health/comorbidities**									
Diabetes	1 (3.4)	9 (1.4)	0.36	0 (0.0)	10 (1.5)	>0.99	0 (0.0)	10 (1.5)	>0.99
Insulin treated diabetes	1 (3.4)	3 (0.5)	0.16	0 (0.0)	4 (0.6)	>0.99	0 (0.0)	4 (0.6)	>0.99
COPD	0 (0.0)	1 (0.2)	>0.99	0 (0.0)	1 (0.2)	>0.99	0 (0.0)	1 (0.1)	>0.99
Obesity	4 (14)	124 (19)	0.63	3 (16)	125 (19)	>0.99	0 (0.0)	128 (19)	0.59
Hypertension	2 (6.9)	24 (3.7)	0.31	2 (11)	24 (3.7)	0.16	0 (0.0)	26 (3.9)	>0.99
Dyspnea	0 (0.0)	3 (0.5)	>0.99	0 (0.0)	3 (0.5)	>0.99	0 (0.0)	3 (0.4)	>0.99
Current smoker	2 (6.9)	45 (7.0)	>0.99	1 (5.3)	46 (7.0)	>0.99	1 (0.0)	46 (6.9)	0.30
Corticosteroid use	0 (0.0)	7 (1.1)	>0.99	0 (0.0)	7 (1.1)	>0.99	0 (0.0)	7 (1.0)	>0.99
Wound infection	0 (0.0)	1 (0.2)	>0.99	0 (0.0)	1 (0.2)	>0.99	0 (0.0)	1 (0.0)	>0.99
**ASA class**			0.27			0.71			0.05
1—No disturbance	12 (41)	347 (54)		10 (53)	349 (53)		1 (20)	358 (54)	
2—Mild disturbance	15 (52)	279 (43)		9 (47)	285 (44)		3 (60)	291 (43)	
3—Severe disturbance	2 (6.9)	19 (2.9)		0 (0.0)	21 (3.2)		1 (20)	20 (3.0)	
4—Life-threatening	0 (0.0)	0 (0.0)		0 (0.0)	0 (0.0)		0 (0.0)	0 (0.0)	
**Wound class**			0.60			0.88			0.46
1—Clean	3 (10)	41 (6.4)		1 (5.3)	43 (6.6)		1 (20)	43 (6.4)	
2—Clean/Contaminated	26 (90)	597 (93)		18 (95)	605 (92)		4 (80)	619 (93)	
3—Contaminated	0 (0.0)	7 (1.1)		0 (0.0)	7 (1.1)		0 (0.0)	7 (1.0)	
4—Dirty/Infected	0 (0.0)	0 (0.0)		0 (0.0)	0 (0.0)		0 (0.0)	0 (0.0)	

**Table 8:** Multivariable assessment of different complication occurrence for all jaw surgery patients.

Complication	OR	95% CI	*p*-Value
**Reoperation**			
Insulin treated diabetes	0.23	0.14–0.31	<0.0001
Outpatient	−0.01	−0.03–0.00	0.04
**Readmission**			
Isolated Triple Jaw Surgery	−0.57	−1.06–−0.08	0.02
Race, Asian	0.43	0.11–0.76	0.009
Obesity Class 2; BMI = 35–39.9	−0.62	−1.11–−0.13	0.01
Outpatient	0.24	0.03–0.45	0.02
**Any surgical complication**			
Isolated Single Lower Jaw Surgery	0.04	0.00–0.08	0.03
Outpatient	0.03	0.00–0.06	0.03
**Superficial Incisional Infection**			
Isolated Single Lower Jaw Surgery	0.04	0.00–0.07	0.04
**Deep Incisional Infection**			
Double Jaw Surgery with concurrent intranasal procedure	0.10	0.06–0.15	<0.0001
**Organ Space Infection**			
Single Jaw Surgery with concurrent genioplasty	0.05	0.03–0.06	0.0002
Hypertension	0.04	0.02–0.06	0.0007
**Bleeding**			
Single Jaw Surgery with concurrent genioplasty	0.05	0.02–0.07	<0.0001
Race, Asian	0.01	0.00–0.02	0.003
**Any medical complication**			
ASA, Class 3	0.05	0.01–0.10	0.01
**DVT**			
Other	0.03	0.01–0.04	0.0002
History of COPD	−0.09	−0.17–−0.02	0.02
Current smoker	0.02	0.01–0.03	0.0007
Underweight; BMI < 18.5	0.03	0.02–0.05	<0.0001
ASA, Class 3	0.06	0.04–0.07	<0.0001
Wound Class 1	0.02	0.01–0.03	0.001

## Data Availability

Restrictions apply to the availability of these data. Data were obtained from the American College of Surgeons—National Surgical Quality Improvement Program. The application can be submitted at https://accreditation.facs.org/programs/nsqip (accessed on 8 February 2023).
